# 3D to 2D Magnetic Ordering of Fe^3+^ Oxides Induced by Their Layered Perovskite Structure

**DOI:** 10.1021/acs.inorgchem.1c00529

**Published:** 2021-05-19

**Authors:** Xabier Martínez de Irujo-Labalde, Ulises Amador, Clemens Ritter, Masato Goto, Midori Amano Patino, Yuichi Shimakawa, Susana García-Martín

**Affiliations:** † Departamento de Química Inorgánica I, Facultad de Ciencias Químicas, Universidad Complutense, 28040 Madrid, Spain; ‡ Inorganic Chemistry Laboratory, Department of Chemistry, University of Oxford, South Parks Road, Oxford OX1 3QR, United Kingdom; § Facultad de Farmacia, Departamento de Química y Bioquímica, Urbanización Montepríncipe, Boadilla del Monte, Universidad San Pablo-CEU, CEU Universities, E-28668 Madrid, Spain; ∥ Institut Laue-Langevin, 6, rue Jules Horowitz, BP 156−38042 Grenoble, Cedex 9, France; ⊥ Institute for Chemical Research, Kyoto University, Uji, Kyoto 611-0011, Japan

## Abstract

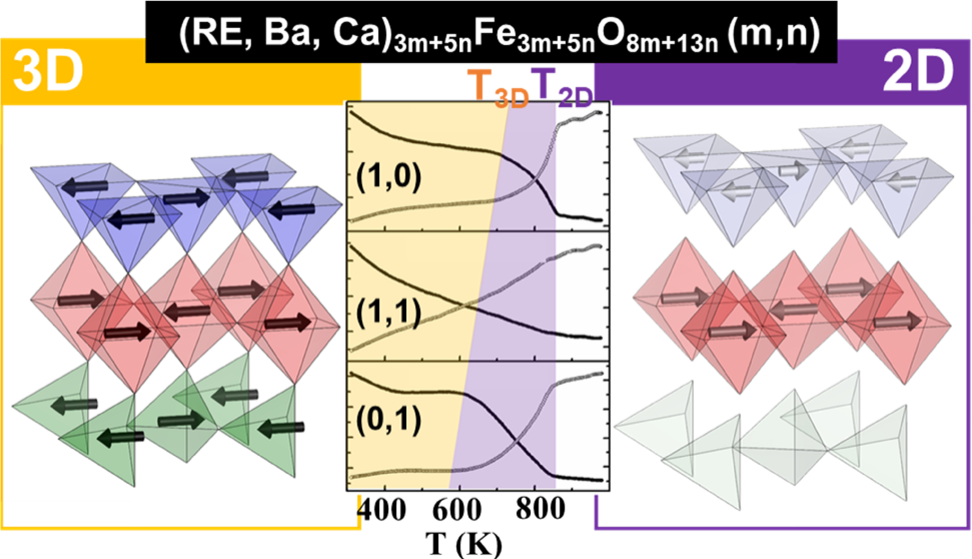

The antiferromagnetic behavior of Fe^3+^ oxides of composition RE_1.2_Ba_1.2_Ca_0.6_Fe_3_O_8_, RE_2.2_Ba_3.2_Ca_2.6_Fe_8_O_21_, and REBa_2_Ca_2_Fe_5_O_13_ (RE = Gd, Tb) is highly influenced by the type of oxygen polyhedron around the Fe^3+^ cations and their ordering, which is coupled with the layered RE/Ba/Ca arrangement within the perovskite-related structure. Determination of the magnetic structures reveals different magnetic moments associated with Fe^3+^ spins in the different oxygen polyhedra (octahedron, tetrahedron, and square pyramid). The structural aspects impact on the strength of the Fe-O-Fe superexchange interactions and, therefore, on the Néel temperature (*T*_N_) of the compounds. The oxides present an interesting transition from three-dimensional (3D) to two-dimensional (2D) magnetic behavior above *T*_N_. The 2D magnetic interactions are stronger within the FeO6 octahedra layers than in the FeO4 tetrahedra layers.

## Abstract 2

The antiferromagnetic behavior of Fe^3+^ oxides is highly influenced by the type of oxygen polyhedron around the Fe^3+^ cations and their ordering, which is coupled with the layered RE/Ba/Ca arrangement, within the perovskite-related structure in this striking (RE, Ba, Ca)_3*m*+5*n*_Fe_3*m*+5*n*_O_8*m*+13*n*_ (*m*, *n*) homologous series.

## Introduction

The variety of perovskite-related crystal structures and properties found in A*_n_*Fe*_n_*O_3*n*–1_ oxides are highly influenced by the nature of A cations. Fe can adopt different coordination polyhedra formed by the oxygen sublattice, mainly octahedra, tetrahedra, and square pyramids, connected in different manners, and these features are partially associated with the type of A atoms. In the case of Fe^3+^ compounds, layered ordering of FeO6 octahedra (O) and FeO4 tetrahedra (T) in a stacking sequence O-T-O-T-O along the [001]_p_ direction (where p refers to the cubic perovskite) yields the so-called Brownmillerite structure with the A_2_Fe_2_O_5_ stoichiometry, adopted by the Sr_2_Fe_2_O_5_ oxide.^1−3^ The Brownmillerite structure is the *n* = 2 member of the A*_n_*Fe*_n_*O_3*n*–1_ homologous series while the perovskite structure, such as that adopted by GdFeO_3_, corresponds to *n* = ∞.^4^ Surprisingly, and probably due to steric effects associated with the A cation, Fe^3+^ with square pyramidal coordination (FeO5, SP), which is generally stable for atoms with the Jahn–Teller distortion, appears in Ba_2_Fe_2_O_5_ ^5^ and some members of the Sr*_n_*Fe*_n_*O_3*n*–1_ series.^6^ The effects of the nature of the A atoms on the Fe-coordination polyhedra are even more evident in quaternary (A/A′)*_n_*Fe*_n_*O_3*n*–1_ systems with different types and ratios of A cations ordered within the crystal structure, usually in a layered manner. For instance, the layered ordering of Y and Ba in YBa_2_Fe_3_O_8_ ^7^ is combined with the layered ordering of FeO5 square pyramids (SP) and FeO6 octahedra (O) in a stacking sequence Y-SP-Ba-O-Ba-SP-Y-SP along [001]_p_. In LaCa_2_Fe_3_O_8_, Fe^3+^ presents octahedral and tetrahedral coordination with La-O-Ca-T-Ca-O-La ordering.^8^ An interesting example is the crystal structure of Ba_2_Ca_2_YFe_5_O_13_ (nominal composition Ba_1.7_Ca_2.4_Y_0.9_Fe_5_O_13_), described as the intergrowth of YBa_2_Fe_3_O_8_ and Ca_2_Fe_2_O_5_ layers, which presents the Fe^3+^ ions in square pyramidal, octahedral, and tetrahedral coordination environments.^9,10^

Furthermore, there is a strong dependence of the electric and magnetic properties of these Fe^3+^ oxides on their crystal structure. Interatomic distances and bonding angles highly impact the Fe-O-Fe magnetic interactions. In fact, bending of the Fe-O-Fe angle implies the appearance of weak ferromagnetism associated with a canting in the spin alignment due to the Dzyaloshinskii–Moriya interactions.^11,12^ This fact combined with the electric polarization that can emerge in the REFeO_3_ orthoferrites have made these materials an interesting source to search for multiferroicity over the last 2 decades.^13,14^ However, little attention has been paid on the implications that the different oxygen polyhedra around the Fe cations can have on the magnetic properties of the Fe perovskites. In this sense, the crystal structure of both LaCa_2_Fe_3_O_8_ and YBa_2_Fe_3_O_8_ favor strong Fe-O-Fe superexchange interactions to sustain antiferromagnetic ordering above room temperature (*T*_N_ = 735 and 660 K, respectively).^8,15^ In principle, different magnetic moments could be expected for Fe^3+^ with different anion environments due to different degrees of covalency of the Fe-O bonding. However, it is not reported like this in general and, in particular in the case of LaCa_2_Fe_3_O_8_ and YBa_2_Fe_3_O_8,_ the magnetic moments of the different Fe atoms in the structure, determined from neutron diffraction results, are found similar despite the fact that the Mössbauer spectrum of LaCa_2_Fe_3_O_8_ shows two different hyperfine parameters associated with different magnetic states.^16^ On the contrary, some studies indicate a relationship between the magnetic moment and the coordination polyhedron of Fe^3+^. In fact, two different magnetic moments associated with Fe^3+^ in octahedral and tetrahedral coordination are clearly distinguished in the recently reported multiferroic Fe oxides with the general formula RE_1.2_Ba_1.2_Ca_0.6_Fe_3_O_8_ (RE = Gd, Tb).^17^

We have found that substitution of RE by Ca in RE_1.2_Ba_1.2_Ca_0.6_Fe_3_O_8_ originates a new A_3*m*+5*n*_Fe_3*m*+5*n*_O_8*m*+13*n*_ homologous series, where A = RE, Ba, and Ca. We have studied the influence of the ordering of the A cations in the type and ordering of the Fe-coordination polyhedra in three compounds of the series with the general formula RE_1.2_Ba_1.2_Ca_0.6_Fe_3_O_8_, RE_2.2_Ba_3.2_Ca_2.6_Fe_8_O_21_, and REBa_2_Ca_2_Fe_5_O_13_ (RE = Gd, Tb), which correspond to the *m* and *n* values 1, 0; 1, 1; and 0, 1, respectively.^17,18^

Here, we report the magnetic structures and the magnetic properties of these compounds. We have found a significant influence of the type and ordering of the Fe-coordination polyhedra on the strength of the superexchange interactions of the oxides. Moreover, we have found a transition from three-dimensional (3D) magnetic ordering to a two-dimensional (2D) magnetic ordering, which is highly influenced by the particular crystal structure of each oxide.

## Experimental Methods

RE_1.2_Ba_1.2_Ca_0.6_Fe_3_O_8_, RE_2.2_Ba_3.2_Ca_2.6_Fe_8_O_21_, and REBa_2_Ca_2_Fe_5_O_13_ (RE = Gd, Tb) perovskite-type oxides were prepared as the *x* = 0, 0.25, and 0.4 members of the system RE_0.8–*x*_Ba_0.8_Ca_0.4+*x*_Fe_2_O_5+δ_^17,18^ by the conventional ceramic method using Gd_2_O_3_ (Aldrich 99.9%), Tb_4_O_7_ (Aldrich 99.9%), BaCO_3_ (Aldrich 99.99%), CaCO_3_ (Merck 99.7%), and Fe_2_O_3_ (Aldrich 99.99%). Y_2_O_3_ was dried at 1173 K prior to weighing to eliminate Y(OH)_2_. Stoichiometric amounts of the starting materials were mixed and heated at 1173 K in air to decompose the Ba and Ca carbonates. Afterward, the samples were pelletized and heated at 1473 K in air for 48 h with intermediate grindings. Initial phase identification and preliminary structural analysis were carried out by powder X-ray diffraction (PXRD) using a Bruker D8 diffractometer.

The nuclear and magnetic structures of the Tb oxides were determined by neutron powder diffraction (NPD) using the D2B and D20 instruments at the ILL (Grenoble, France). High-resolution diffraction patterns were collected on D2B at 300 and 1000 K using a wavelength λ = 1.594 Å. To determine the thermal evolution of the magnetic structure, patterns were collected between 300 and 1000 K every 3.5 K on D20 with neutrons of λ = 2.41 Å, using a quartz tube open to air inside a furnace under vacuum. The data were fitted using FullProf.^19^ Magnetic symmetry analysis was done using the program BASIREPS.^20,21^

^57^Fe Mössbauer spectra were measured to confirm the oxidation states and oxygen environments of the Fe cations, and to study the magnetic states in the compounds. The measurements were performed at room temperature in transmission geometry with a constant acceleration spectrometer using a ^57^Co/Rh radiation source. The velocity scale and the isomer shift (IS) were determined with the relative values of α-Fe at RT. The spectra were fitted to Lorentzian functions using the standard least-squares method.

The magnetic properties of the compound were measured with a CS-3 furnace apparatus at 5 Oe in the temperature range between 300 and 950 K. Hysteresis loops at 300 and 2 K were collected in a Quantum Design MPMS-XL SQUID spectrometer in the range between 50 and −50 kOe.

## Results and Discussion

RE_1.2_Ba_1.2_Ca_0.6_Fe_3_O_8_, RE_2.2_Ba_3.2_Ca_2.6_Fe_8_O_21_, and REBa_2_Ca_2_Fe_5_O_13_ (RE = Gd, Tb) oxides have a layered perovskite-type structure. The Tb and Gd compounds are isostructural. The crystal structures have been determined by transmission electron microscopy techniques with atomic resolution in the Gd oxides.^17,18^ The nuclear structures have been confirmed and refined in the Tb oxides by means of neutron powder diffraction (NPD) experiment data collected at 300 and 1000 K. The results of the structure refinements are collected in the Supporting Information (Figure S1 and Tables S1–5), and in ref (17). Figure S2 displays the representation of the building blocks of the crystal structures of the three compounds. Analogous to the previously reported structure of Gd compounds,^17,18^ these crystal structures consist of layered ordering of the RE^3+^, Ba^2+^, and Ca^2+^ cations in combination with layered ordering of the different coordination polyhedra of the Fe atoms along the stacking *c*-axis. Substitution of RE by Ca in the systems modifies the layered ordering of these cations and the oxygen content in such a way that the RE^3+^, Ba^2+^, and Ca^2+^ ordering is established in blocks of 3*a*_p_ periodicity in the RE_1.2_Ba_1.2_Ca_0.6_Fe_3_O_8_ oxides; in blocks of 5*a*_p_ periodicity in the REBa_2_Ca_2_Fe_5_O_13_ oxides; and by intercalation of blocks of 3*a*_p_ and 5*a*_p_ periodicity in the intermediate RE_2.2_Ba_3.2_Ca_2.6_Fe_8_O_21_ compounds.^17,18^ The different RE^3+^, Ba^2+^, and Ca^2+^ ordering drives the formation of the different Fe-coordination polyhedra (tetrahedron, octahedron, and square pyramid) and their different layered ordering leading to the current structures.^17,18^

The Tb_1.2_Ba_1.2_Ca_0.6_Fe_3_O_8_ oxide presents a layered-type perovskite structure with a √2*a*_p_ × √2*a*_p_ × 3*a*_p_ unit cell related to the layered ordering of A cations in a Tb/Ca-Tb/Ca-Ba-Tb/Ca sequence with a combined T-O-O-T ordering sequence of the Fe-O polyhedra. The crystal structure of Tb_2.2_Ba_3.2_Ca_2.6_Fe_8_O_21_ presents an 8-fold layered ordering of the A atoms in a stacking sequence Tb/Ca-Tb/Ca-Ba-Tb-Ba-Tb/Ca-Tb/Ca-Ba-Tb/Ca along the *c*-axis, combined with a sequence of oxygen environments around the Fe atoms T_L_-(O)_2_-T_L_-O-(SP)_2_-O-T_R_-(O)_2_-T_R_-O-(SP)_2_-O-T_L_ (T_L_ and T_R_ denote two different orientations (left and right) of the tetrahedra); the additional ordering of tetrahedra chains running along the *a*-axis gives rise to a 16-fold ordering and a unit cell √2*a*_p_ × √2*a*_p_ × 16*a*_p_. The crystal structure of TbBa_2_Ca_2_Fe_5_O_13_ is perovskite-related with a √2*a*_p_ × √2*a*_p_ × 10*a*_p_ unit cell due to layered ordering of Tb, Ba, and Ca (stacking sequence Tb/Ca-Tb/Ca-Ba-Tb-Ba-Tb/Ca) in combination with the T_L_-O-(SP)_2_-O-T_R_-O-(SP)_2_-O-T_L_ ordering sequence along the *c*-axis of the oxygen coordination polyhedra of the Fe atoms. Finally, it is worthy to note, some disorder is observed in the oxygen atoms shared along the tetrahedra chains in both the TbBa_2_Ca_2_Fe_5_O_13_ and Tb_2.2_Ba_3.2_Ca_2.6_Fe_8_O_21_ oxides, but the R or L orientation of the chains is preserved.

The stoichiometry of these compounds agree with the formulation of the new A_3*m*+5*n*_Fe_3*m*+5*n*_O_8*m*+13*n*_ homologous series (A = Gd^3+^ or Tb^3+^, Ba^2+^, Ca^2+^) with *m* = 1, *n* = 0 for RE_1.2_Ba_1.2_Ca_0.6_Fe_3_O_8_; *m* = 1, *n* = 1 for RE_2.2_Ba_3.2_Ca_2.6_Fe_8_O_21_; and *m* = 0, *n* = 1 for REBa_2_Ca_2_Fe_5_O_13_. These RE_1.2_Ba_1.2_Ca_0.6_Fe_3_O_8_ oxides are multiferroic, exhibiting antiferromagnetic properties and a polar structure with a moment of 33, 0 μC/cm^2^ in the case of the Gd compound and 23, 2 μC/cm^2^ in the Tb one.^17^ Interestingly, there are three different coordination polyhedra of the Fe atoms in the crystal structure of REBa_2_Ca_2_Fe_5_O_13_. This uncommon structural feature has only previously been found to be unstable in air in the Nd_2_Ba_2_Ca_2_Fe_6_O_15_ oxide^22^ and in nanodomains of some compounds of the RE_2–*x*_Ba_3+*x*_Fe_5–*x*_Co*_x_*O_15-δ_ (TR = Y, Pr, Nd, Sm y Gd) systems.^23−26^

**Figure 1:**
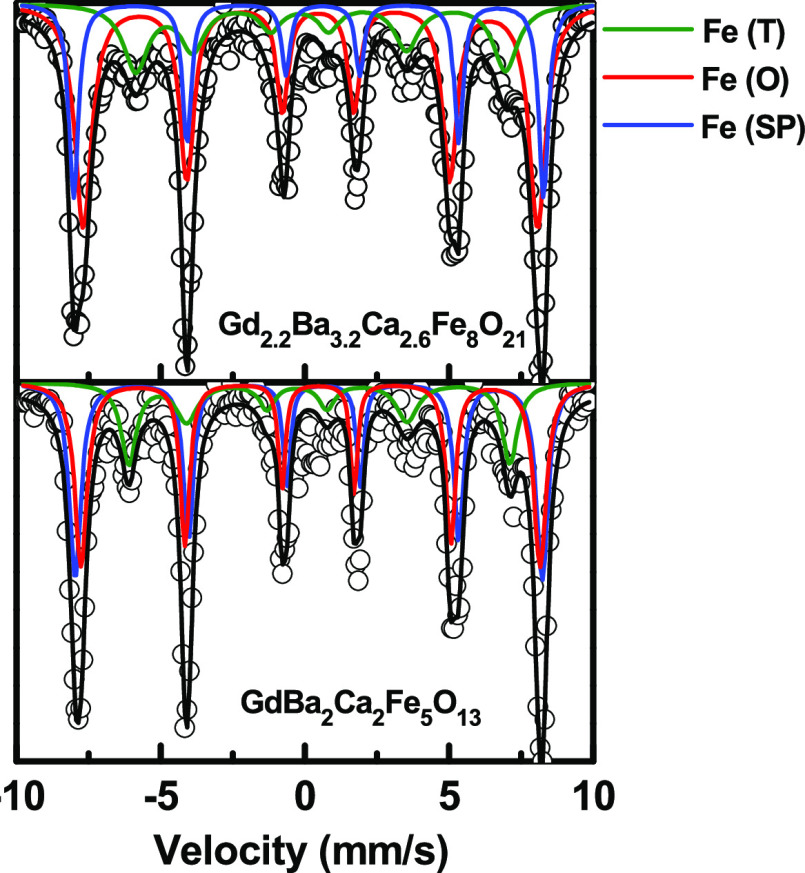
Mössbauer spectra of the Gd_2.2_Ba_3.2_Ca_2.6_Fe_8_O_21_ and GdBa_2_Ca_2_Fe_5_O_13_ oxides collected at room temperature. The spectrum of Gd_1.2_Ba_1.2_Ca_0.6_Fe_3_O_8_ is found in ref (17).

The Mössbauer spectra of these oxides are consistent with different Fe^3+^ coordination sites and their corresponding magnetic ordering within the crystal structure. EELS analysis also agrees with Fe^3+^.^17,18^Figure 1 shows the Mössbauer spectra of Gd_2.2_Ba_3.2_Ca_2.6_Fe_8_O_21_ and GdBa_2_Ca_2_Fe_5_O_13_ collected at room temperature and Table 1 summarizes the corresponding IS, QS, and HF parameters. The spectrum of GdBa_2_Ca_2_Fe_5_O_13_ consists of three different components in an area ratio of 1:2:2; two of them with IS and QS values attributed to Fe^3+^ sites in octahedral and tetrahedral coordination like in G_1.2_Ba_1.2_Ca_0.6_Fe_3_O_8_ ^17^ and a third component with IS and QS values of 0.39 and −0.53 mm/s, respectively, associated with Fe^3+^ in the square pyramidal environment. All of the components are split into six lines, indicating magnetic ordering of Fe^3+^ within the crystal structure, also like in Gd_1.2_Ba_1.2_Ca_0.6_Fe_3_O_8_.^17^^17^ The Mössbauer spectrum of Gd_2.2_Ba_3.2_Ca_2.6_Fe_8_O_21_ presents three different components in an area ratio of 2:4:2 attributed to Fe^3+^ sites in tetrahedral, octahedral, and square pyramidal coordination, respectively, and also splits into six lines due to the magnetic ordering of Fe^3+^ within the crystal structure. In addition to the Mössbauer spectra results, the appearance of magnetic reflections in the neutron diffraction patterns of the Tb oxides indicate magnetic ordering below 700 K, as we will comment.

**Table 1:** Parameters for the Fitting of the Room Temperature Mössbauer Spectra of Gd_2.2_Ba_3.2_Ca_2.6_Fe_8_O_21_ (16) and Gd_2_Ba_2_CaFe_5_O_13_ (10)

	component	IS (mm/s)	QS (mm/s)	HF (T)	%
16	Fe (T)	0.20(4)	0.70(7)	39.9(3)	25
	Fe (O)	0.33(1)	–0.28(2)	48.8(1)	50
	Fe (SP)	0.37(1)	–0.49(2)	50.6(1)	25
10	Fe (T)	0.12(3)	0.76(6)	41.0(1)	20
	Fe (O)	0.32(1)	–0.25(2)	49.4(1)	40
	Fe (SP)	0.39(1)	–0.53(2)	50.1(1)	40

**Figure 2:**
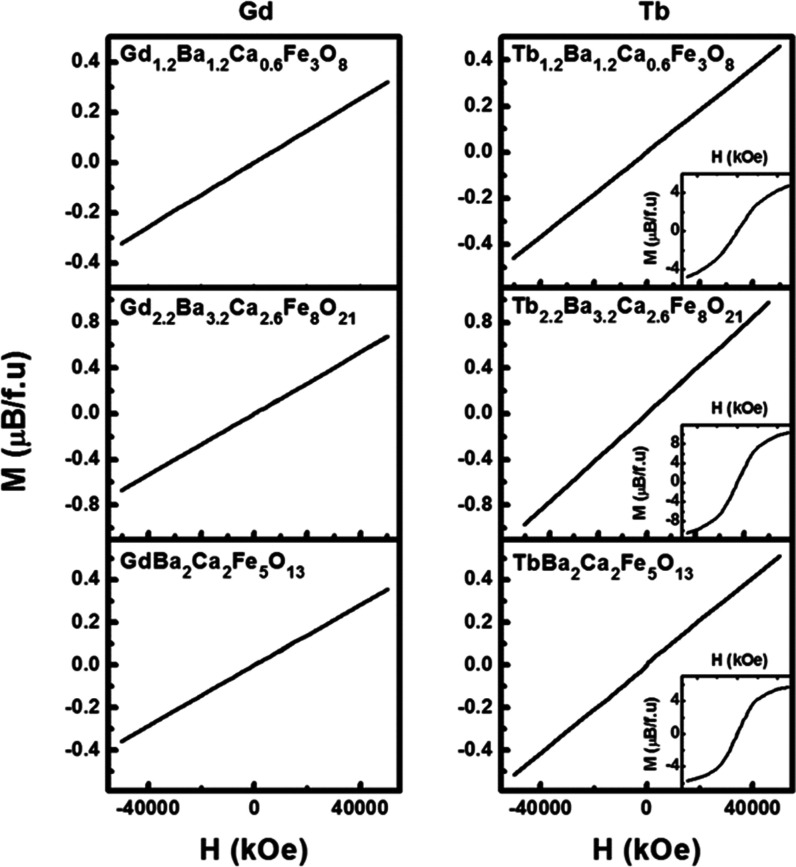
Magnetization (M) as a function of applied field (H) of the RE_1.2_Ba_1.2_Ca_0.6_Fe_3_O_8_, RE_2.2_Ba_3.2_Ca_2.6_Fe_8_O_21_, and REBa_2_Ca_2_Fe_5_O_13_ oxides with RE = Gd (left) and Tb (right) collected at room temperature. The insets show the M–H curves of the Tb oxides at 2k.

Figure 2 displays the magnetization vs magnetic field data of both the Gd and Tb compounds collected at 300 K. In both cases the magnetization is linear up to 5 T showing neither remanet magnetization nor a coercive field, which suggests antiferromagnetic ordering of Fe^3+^ (*d*^5^ in high spin, *S* = 5/2) within the structure. Despite the tilting of the oxygen octahedra and the deviation from 180° of the Fe-O-Fe angle within the tetrahedral layer, signs of ferromagnetic behavior associated with the Dzyaloshinskii–Moriya interactions are not observed. The possibility of the Fe^3+^-FM behavior being screened by the high values of paramagnetic Tb^3+^ and Gd^3+^ components cannot be excluded; however, the implications of these interactions are beyond the scope of the present work. The “S” shape of the magnetic cycles at 2k of the Tb oxides are probably related to the combination of the Brillouin curve of paramagnetic Tb^3+^ cations and linear contribution of antiferrromagnetically ordered Fe^3+^ atoms.

**Figure 3:**
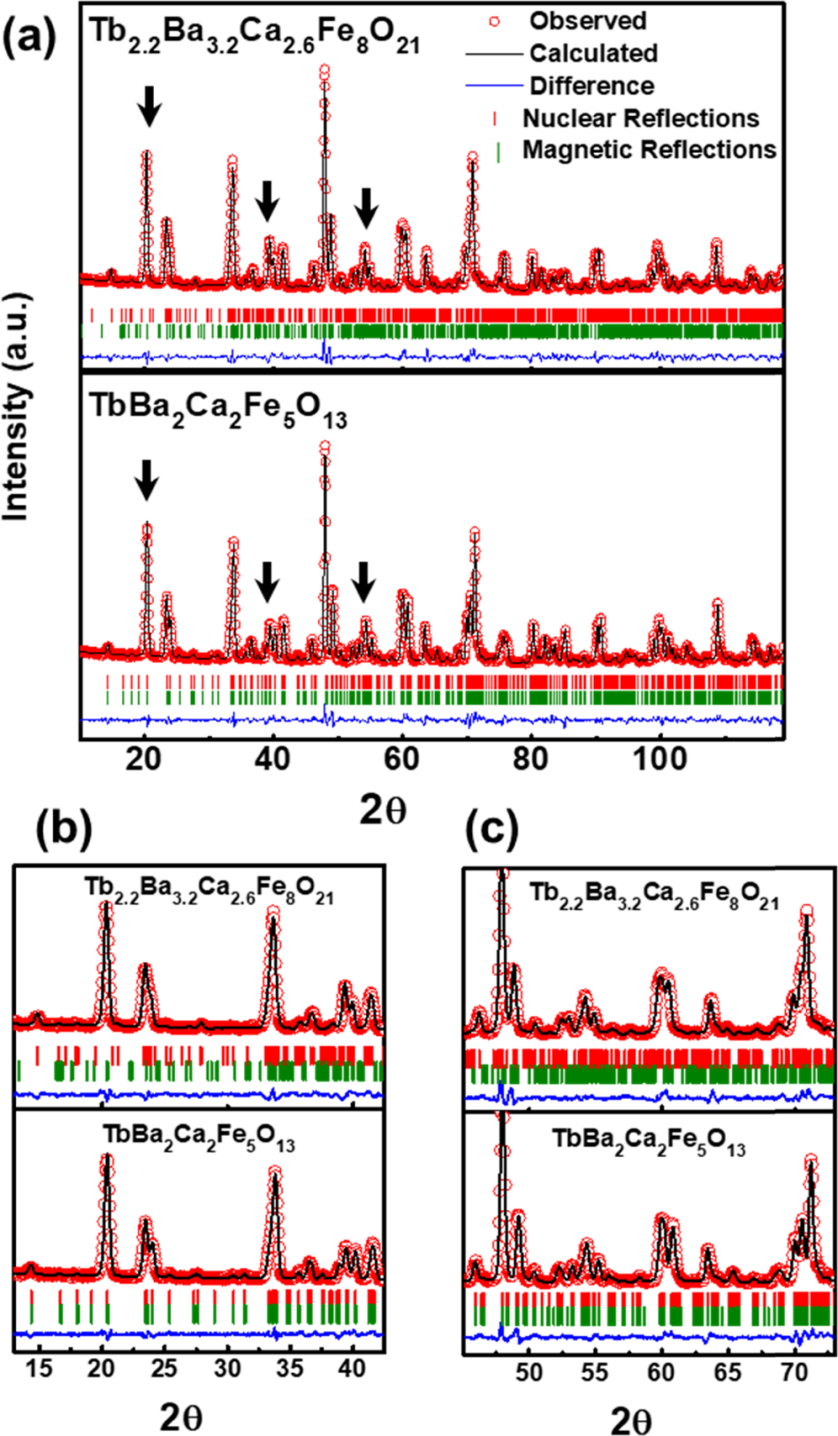
(a) Experimental and calculated NPD patterns at room temperature of Tb_2.2_Ba_3.2_Ca_2.6_Fe_8_O_21_ and TbBa_2_Ca_2_Fe_5_O_13_. Green bars indicate the positions of nuclear (upper row) and magnetic (lower row) Bragg peaks. The strongest magnetic peaks are indicated by arrows. (b) and (c) Selected 2θ regions of the NPD patterns depicted in (a). The patterns of Tb_1.2_Ba_1.2_Ca_0.6_Fe_3_O_8_ can be found in ref (17).

**Figure 4:**
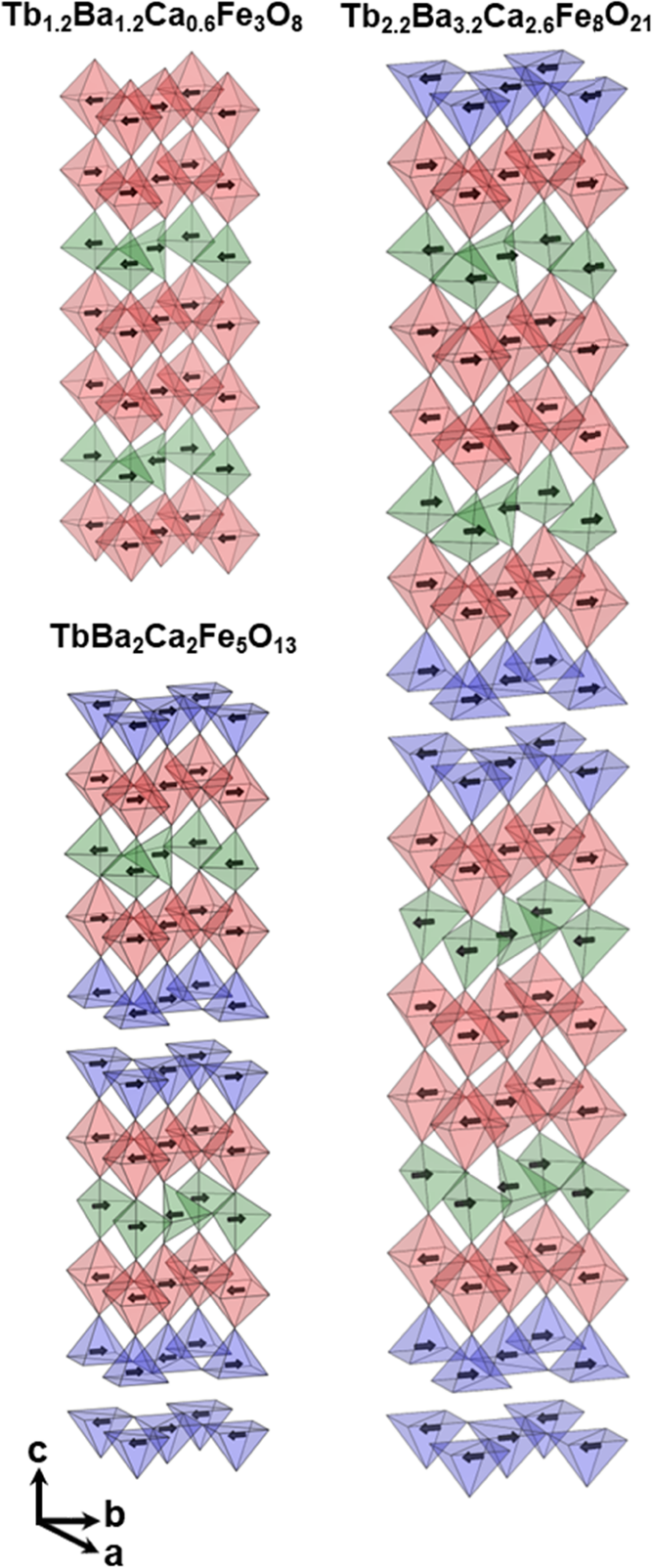
Magnetic structures of the Tb_1.2_Ba_1.2_Ca_0.6_Fe_3_O_8_, Tb_2.2_Ba_3.2_Ca_2.6_Fe_8_O_21_, and TbBa_2_Ca_2_Fe_5_O_13_ oxides.

NPD patterns of the Tb compounds collected at 300 and 1000 K were used to determine the magnetic structures. Magnetic reflections in the NPD patterns appear below 700 K (Figure 3). In the case of Tb_1.2_Ba_1.2_Ca_0.6_Fe_3_O_8_, the magnetic reflections were indexed by a propagation vector [0 0 1/2] producing the magnetic unit cell √2*a*_p_ × √2*a*_p_ × 6*a*_p_.^17^ The magnetic reflections are coincident with nuclear ones ([000] propagation vector) in the diffraction pattern of TbBa_2_Ca_2_Fe_5_O_13_, indicating nuclear and magnetic unit cells with similar dimensions (√2*a*_p_ × √2*a*_p_ × 10*a*_p_). In the pattern of Tb_2.2_Ba_3.2_Ca_2.6_Fe_8_O_21_, the magnetic reflections are indexed with the [0 1 0] propagation vector yielding the √2*a*_p_ × √2*a*_p_ × 16*a*_p_ magnetic unit cell. Figure 4 shows the magnetic structures of the three different oxides. The three magnetic structures consist of a three-dimensional G-type antiferromagnetically ordered arrangement of the Fe spins, with the spin directions lying along the *b*-axis. At room temperature, two different magnetic moments of 3.1(2) μB and 4.04(9) μB for Fe^3+^ spins in tetrahedral and octahedral sites (respectively) are determined in the Tb_1.2_Ba_1.2_Ca_0.6_Fe_3_O_8_.^17^ Three magnetic moments associated with Fe^3+^ spins in tetrahedral, octahedral, and square pyramidal coordination sites are observed for both TbBa_2_Ca_2_Fe_5_O_13_ and Tb_2.2_Ba_3.2_Ca_2.6_Fe_8_O_21_. For the former oxide, values of 3.0(3), 3.8(2), and 3.9(2) μB correspond to Fe^3+^ in tetrahedral, octahedral, and square pyramidal sites (respectively). For Tb_2.2_Ba_3.2_Ca_2.6_Fe_8_O_21_, magnetic moments of 3.3(3), 4.0(2), and 4.2(3) μB are determined for analogous sites.

The theoretical value of the magnetic moment of Fe^3+^ (*d*^5^ in high spin, *S* = 5/2) is 5 μB. The lower values observed can be attributed to the covalence of the Fe-O bonding, which is comparatively higher when Fe^3+^ locates at tetrahedral sites than at octahedral or square pyramidal sites, and also to thermal spin fluctuations. The magnetic moment values agree with those calculated from the HF parameters of the Mössbauer spectra of the corresponding Gd oxides. Therefore, these results clearly demonstrate the relationship between the magnetic moment and the coordination polyhedra of the Fe atoms, probably due to differences in the nature of the Fe-O bonding.

**Figure 5:**
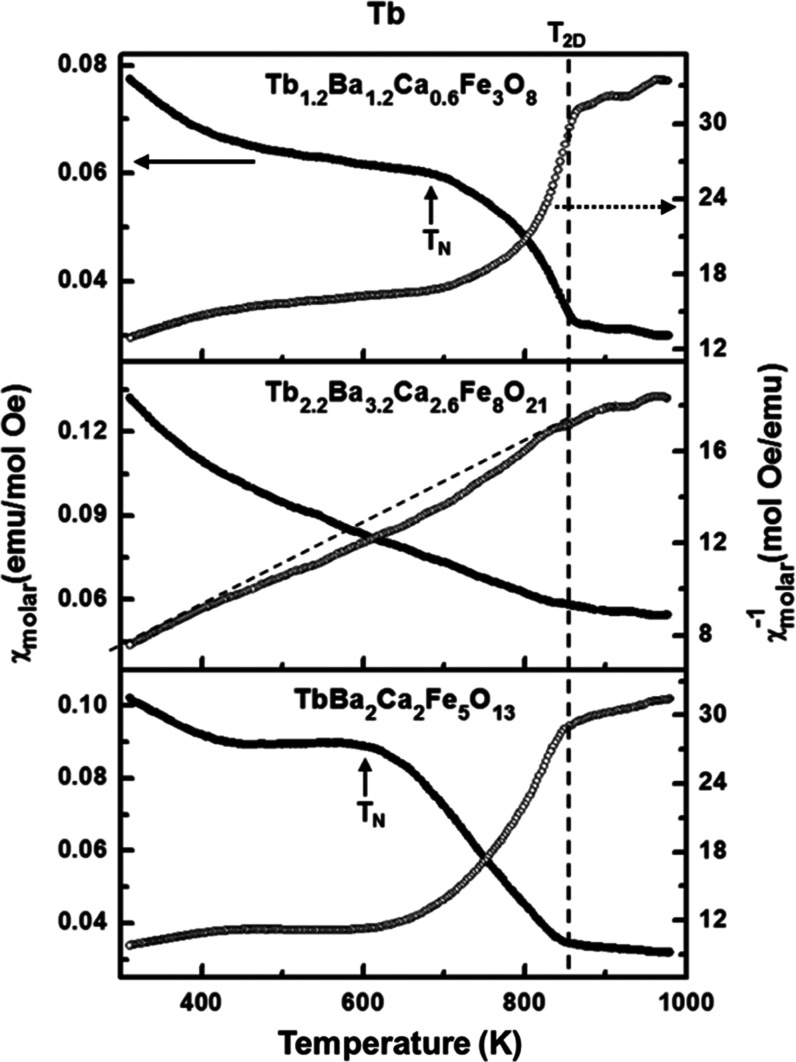
Thermal evolution of the magnetic susceptibility (left axis) and its inverse (right axis) under a magnetic field of 5 Oe in the range between 300 and 1000 K of the Tb_1.2_Ba_1.2_Ca_0.6_Fe_3_O_8_, Tb_2.2_Ba_3.2_Ca_2.6_Fe_8_O_21_, and TbBa_2_Ca_2_Fe_5_O_13_ oxides.

The dependence of the magnetic susceptibility with the temperature reveals interesting and deeper information not only about the temperature range of the magnetic ordering of these compounds, but also regarding their magnetic behavior in relation to the crystal structure. Figure 5 shows the variation of the magnetic susceptibility of Tb oxides with temperature under a magnetic field of 5 Oe in the range between 300 and 1000 K (the Gd oxides ones are collected in Figure S3). Antiferromagnetic ordering is maintained well above room temperature due to the strong Fe-O-Fe superexchange interactions. All of the compounds show broad magnetic transitions in which two transition temperatures are clearly distinguished. The highest transition temperature is similar for all of the Gd oxides (∼910 K) as well as for the Tb oxides (∼850 K). However, the lower transition temperature, considered at the inflection point of the magnetic susceptibility and called the Néel temperature, seems to depend on the compound and therefore on its crystal structure. For a better understanding of this peculiar behavior, we have compared the temperature dependence of the magnetic susceptibility with the variation of the magnetic moments determined from the neutron diffraction experiments collected at different temperatures. For this, we have chosen Tb_1.2_Ba_1.2_Ca_0.6_Fe_3_O_8_ because the differences between the magnetic moments of Fe atoms in octahedral and tetrahedral sites are large while those of Fe atoms in square pyramidal sites are close to the magnetic moments of Fe atoms in octahedral ones.

**Figure 6:**
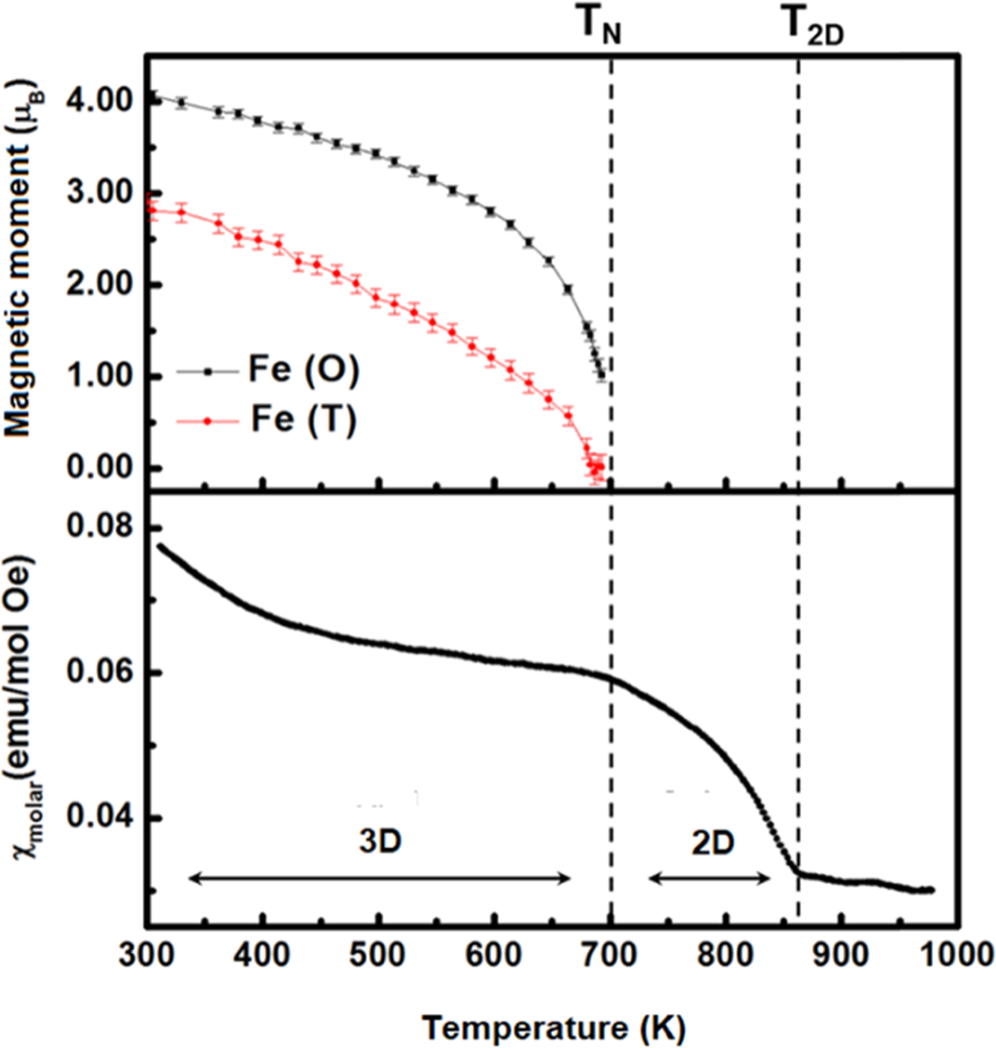
Thermal evolution of the magnetic moment (upper panel) of the Fe atoms in the two different coordination polyhedra (octahedra and tetrahedra) in Tb_1.2_Ba_1.2_Ca_0.6_Fe_3_O_8_ and of its magnetic susceptibility (lower panel).

Figure 6 shows a comparison between the refined magnetic moment for Fe atoms in the two different coordination polyhedra (octahedra and tetrahedra), and the variation of the magnetic susceptibility, both parameters as a function of temperature. Two regions are observed in the magnetic susceptibility data. We interpret these results as being a consequence of the presence of a three-dimensional (3D) type of magnetic ordering as well as of a bidimensional (2D) type of magnetic ordering. The 3D antiferromagnetic ordering is maintained up to the Néel temperature. At higher temperatures, between the Néel temperature (*T*_3D_ = ∼700 K) and the temperature of the second magnetic transition (∼850 K as determined by magnetic susceptibility measurements), the lowering of the total magnetic moment is associated with the loss of 3D magnetic ordering, while the 2D magnetic ordering is maintained up to the so-called *T*_2D_. The drop of the total magnetic moment that takes place between *T*_N_ and *T*_2D_ agrees with the 3D magnetic ordering being lost, while the 2D magnetic ordering is maintained up to *T*_2D_. In this context, *T*_N_ can be referred to as *T*_3D_. Within this temperature range, although the magnetic reflections in the NPD patterns disappear (Figure S4), some magnetic scattering remains as a signature of the 2D ordering until it disappears above *T*_2D_ (Figure S5). This type of 2D and 3D magnetic regions have also been observed in Ca_2_FeMnO_5_^27^ and RuSr_2_GdCu_2_O_8_,^28^ both with perovskite-related structures and layered ordering of Fe or Ru in different oxygen environments.

**Figure 7:**
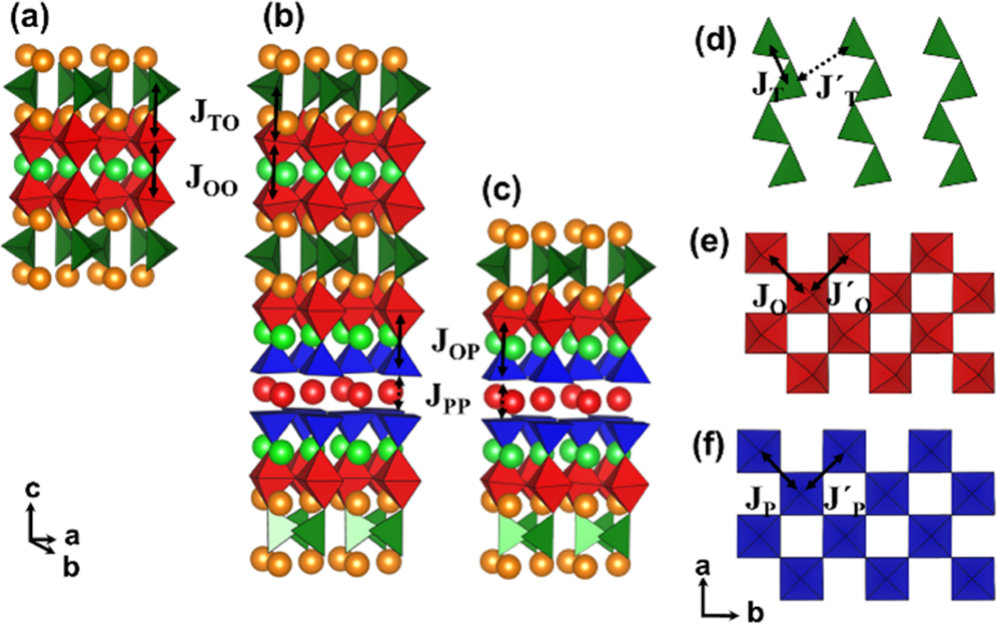
Superexchange interactions of Fe-O-Fe within the structures of RE_1.2_Ba_1.2_Ca_0.6_Fe_3_O_8_, RE_2.2_Ba_3.2_Ca_2.6_Fe_8_O_21_, and REBa_2_Ca_2_Fe_5_O_13_ (a–c) interlayer paths and (d–f) intralayer paths.

The 3D and 2D magnetic behavior can be interpreted in terms of the different strengths of the Fe-O-Fe superexchange interactions within the structure, which are summarized in Figure 7. Interestingly, the sustainable long Fe-O-Fe distance through the apical oxygen connecting the Fe tetrahedra and Fe octahedra (∼2.2 Å at 300 K) can be associated with a weaker superexchange interaction (denoted as *J*_OT_ in Figure 7) due to a weaker overlap between the Fe 3d and O 2p orbitals within this region. When the temperature increases, this *J*_TO_ superexchange interaction weakens enough to be suppressed, while all of the strong interactions within the octahedral blocks (the intralayer *J*_O_ and *J*′_O_, and the interlayer *J*_OO_ interactions) and within the tetrahedral layers (*J*_T_ interactions) still remain. The extremely weak *J*′_T_ interaction between the Fe-tetrahedra chains establishes a pseudo one-dimensional (1D) chain magnetic ordering in the tetrahedral layers, which in fact cannot lead to a long-range ordering, analogous to Na_2_FeSbO_5_, which shows a spin glassy state at very low temperatures.^29^ Thus, the magnetic moment of the tetrahedral layers is mainly related to the *J*_TO_ superexchange interactions, which above the *T*_N_ temperature are not strong enough and explain why the magnetic moment is only preserved within the octahedral blocks above the *T*_N_. The FeO6 octahedra seem to provide the 3D magnetic coupling between layers of Fe atoms in different coordination polyhedra, each of them with a 2D character because the FeO6 layers present not only a higher number of superexchange Fe-O-Fe interaction pathways but also these interactions are stronger due to the shorter Fe-O-Fe distances (∼1.97 Å in average at 300 K). Therefore, the peculiar magnetic behavior in Tb_1.2_Ba_1.2_Ca_0.6_Fe_3_O_8_ is intimately connected with the layered stacking of the two different oxygen polyhedra, octahedra, and tetrahedra.

At this point, the influence of the crystal structure on the 3D magnetic ordering is worth noting: the RE_1.2_Ba_1.2_Ca_0.6_Fe_3_O_8_ oxides present the highest *T*_N_ values (*T*_N_ ∼ 790 K in Gd compounds and *T*_N_ ∼ 700 K in Tb compounds), whereas the 3D magnetic transition occurs at lower temperatures in the REBa_2_Ca_2_Fe_5_O_13_ oxides (*T*_N_ ∼ 700 K in Gd compounds and *T*_N_ ∼ 625 K in Tb compounds). Determination of the *T*_N_ from the magnetic susceptibility data is difficult in the case of the RE_2.2_Ba_3.2_Ca_2.6_Fe_8_O_21_. The *T*_N_ reduction in REBa_2_Ca_2_Fe_5_O_13_ can be explained by the introduction of the pyramidal Fe bilayer in between the octahedral Fe layers. Within this bilayer, the interlayer *J*_PP_ interaction between the Fe cations is negligible due to the loss of the apical oxygen, which creates a bidimensional disruption in the tridimensional magnetic ordering and explains the drop in the *T*_N_ of REBa_2_Ca_2_Fe_5_O_13_ with respect to RE_1.2_Ba_1.2_Ca_0.6_Fe_3_O_8_. In the case of RE_2.2_Ba_3.2_Ca_2.6_Fe_8_O_21,_ the presence of a smaller number of pyramidal Fe bilayer per unit cell would suggest an intermediate *T*_N_ between the other two oxides, but a more detailed study is needed.

Interestingly, in the temperature range between *T*_3D_ (*T*_N_) and *T*_2D_ only Fe^3+^ cations in particular bidimensional regions contribute to the magnetic ordering. As previously mentioned, only the Fe^3+^ cations interacting in more than one direction with other Fe^3+^ cations can contribute to the magnetic ordering. Thus, the superexchange interactions within the pyramidal and octahedral layers in the three types of structures studied here are responsible for the magnetic ordering in this region. The similar strength of the interactions can explain the observed independence of *T*_2D_ as a function of composition.

Therefore, we have identified the major role of the type of the oxygen polyhedra and their stacking over the Fe-O-Fe superexchange interactions.

## Conclusions

The RE_1.2_Ba_1.2_Ca_0.6_Fe_3_O_8_, RE_2.2_Ba_3.2_Ca_2.6_Fe_8_O_21_, and REBa_2_Ca_2_Fe_5_O_13_ (RE = Gd, Tb) perovskite-type oxides of the new A_3*m*+5n_Fe_3*m*+5*n*_O_8*m*+13*n*_ homologous series (A = Gd^3+^ or Tb^3+^, Ba^2+^, Ca^2+^ with *m* = 1, *n* = 0; *m* = 1, *n* = 1; *m* = 0, *n* = 1, respectively), present three-dimensional G-type antiferromagnetic ordering of the Fe^3+^ spins.

The RE/Ba/Ca composition highly influences the layered ordering of these cations as well as the type and layered ordering of the oxygen polyhedra around the Fe^3+^ cations of these oxides, which in return determines their magnetic behavior. These antiferromagnetic compounds show a 3D magnetic ordering below *T*_N_ with a transition to a 2D magnetic ordering maintained within the range *T*_N_ < *T* < *T*_2D_. The transition temperature *T*_N_, which depends on the strength of the Fe-O-Fe superexchange interactions, is clearly associated with the crystal structure of the compounds. The strongest 3D superexchange interactions are established between FeO6 octahedra bilayers through the apical oxygen. This explains why the RE_1.2_Ba_1.2_Ca_0.6_Fe_3_O_8_ oxides show the highest *T*_N_, as they contain the highest number of FeO6 octahedra bilayers per unit cell among the three systems. The 2D magnetic interactions are certainly stronger within the FeO6 octahedra layers than in the FeO4 tetrahedra layers. Therefore, control in the composition of the A_3*m*+5*n*_Fe_3*m*+5*n*_O_8*m*+13*n*_ (A = RE, Ba, Ca) compounds could lead to a very precise tuning of the magnetic properties in this type of systems.

## Supporting Information Available

NPD patterns at 1000 K; crystal structure plots; thermal evolution of the magnetic susceptibility (Gd oxides); variable temperature NPD patterns;, evolution of the intensity of selected magnetic reflections as a function of temperature; refined crystal structure parameters at 300 and 1000 K; and Fe-O distances at 300 and 1000 K (PDF)
