# Supplementary material for: 3D to 2D Magnetic Ordering of Fe3+ Oxides Induced by Their Layered Perovskite Structure
Source: Inorg Chem. 2021 May 19;60(11):8027–34. doi: 10.1021/acs.inorgchem.1c00529 (PMC8478276; doi:10.1021/acs.inorgchem.1c00529)
Supplement: Supplementary file 1 — ic1c00529_si_001.pdf [file ic1c00529_si_001.pdf]

SUPPORTING INFORMATION

3D to 2D magnetic ordering of Fe^{3+}-oxides induced by their layered-perovskite structure

Xabier Martínez de Irujo-Labalde[†,‡], Ulises Amador[§], Clemens Ritter[‖], Masato Goto[#], Midori Amano Patino[#], Yuichi Shimakawa[#] and Susana García-Martín[†,*]

[†]Departamento de Química Inorgánica I, Facultad de Ciencias Químicas, Universidad Complutense, 28040 Madrid, Spain

[‡]Inorganic Chemistry Laboratory, Department of Chemistry, University of Oxford, South Parks Road, Oxford OX1 3QR, United Kingdom

[§]Universidad San Pablo-CEU, CEU Universities, Facultad de Farmacia, Departamento de Química y Bioquímica, Urbanización Montepríncipe, Boadilla del Monte, E-28668, Madrid, Spain

[‖]Institut Laue-Langevin, 6, rue Jules Horowitz, BP 156–38042, Grenoble, Cedex 9, France

[#]Institute for Chemical Research, Kyoto University, Uji, Kyoto 611-0011, Japan

Figures

Figure S1	NPD patterns at 1000 K
Figure S2	Crystal structure plots
Figure S3	Thermal evolution of the magnetic susceptibility (Gd-oxides)
Figure S4	Variable temperature NPD pattern
Figure S5	Evolution of the intensity of selected magnetic reflections as af function of temperature

Tables

Table S1	Refined crystal structure parameters of at 300 and 1000 K.
Table S2	Fe-O distances at 300 and 1000 K

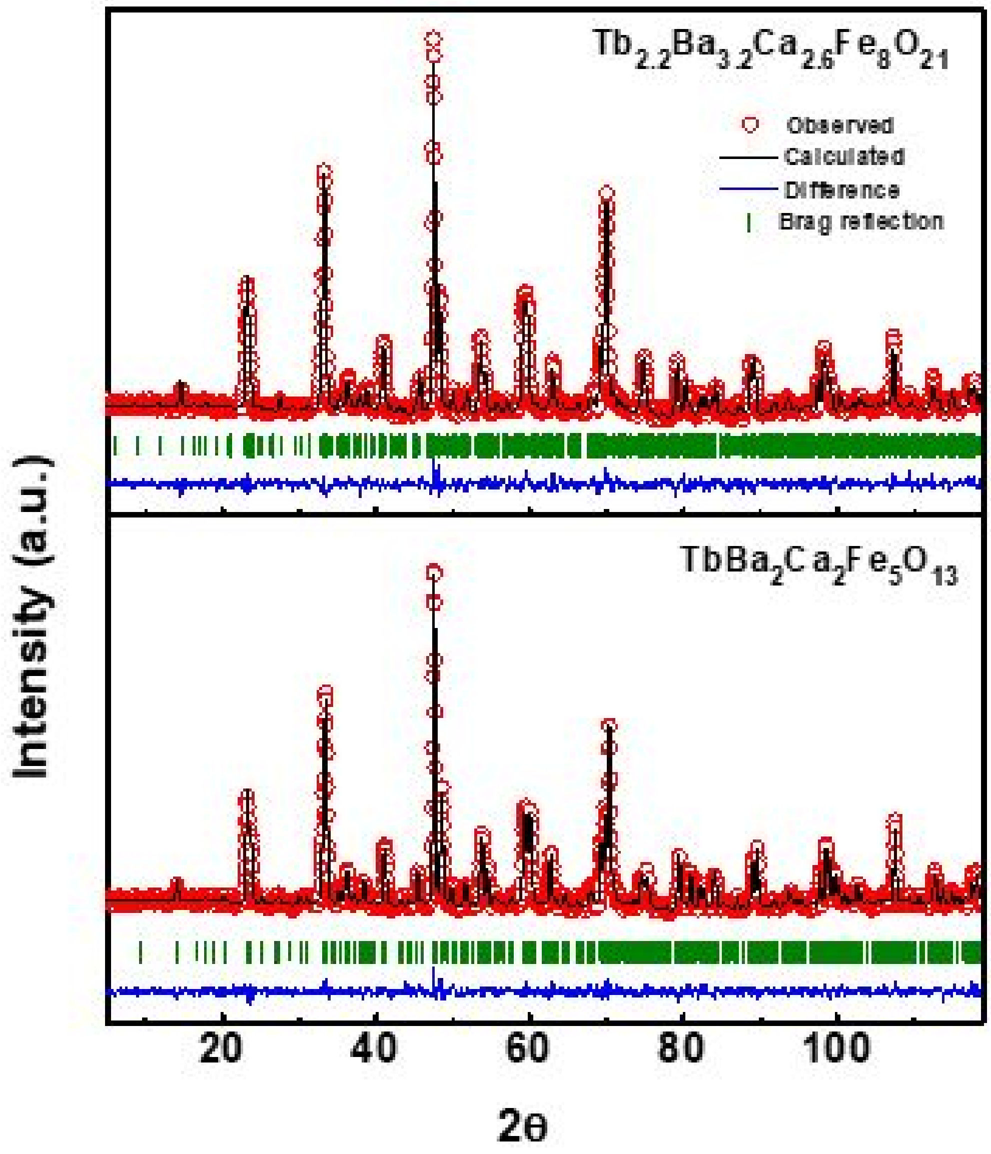

Figure. S1. Rietveld Refinement of the NPD patterns of $Tb_{2.2}Ba_{3.2}Ca_{2.6}Fe_8O_{21}$ and $TbBa_2Ca_2Fe_5O_{13}$ taken at 1000 K in the paramagnetic domain; the observed (red) and calculated (black) patterns, and their difference (blue line in the bottom), are shown. Vertical bars indicate the positions of Bragg peaks.

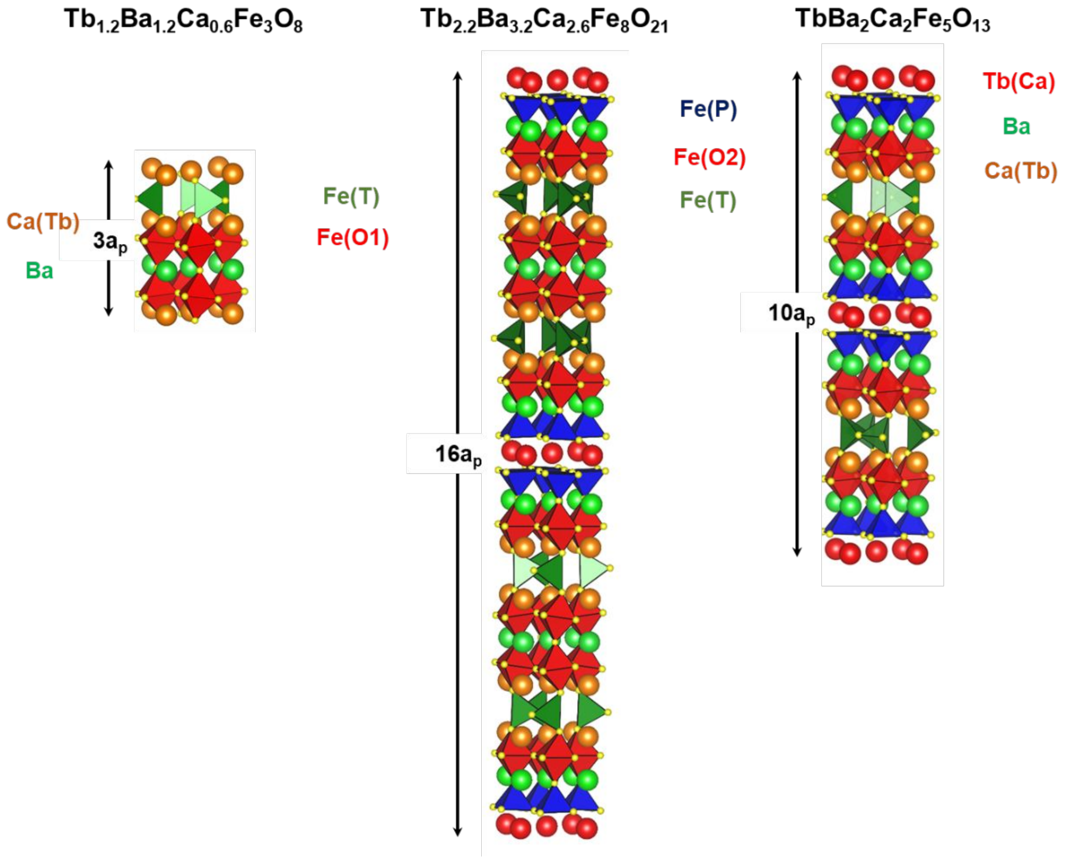

Figure S2. Graphic representation of the nuclear crystal structures of the compounds $Tb_{1.2}Ba_{1.2}Ca_{0.6}Fe_3O_8$, $Tb_{2.2}Ba_{3.2}Ca_{2.6}Fe_8O_{21}$ and $TbBa_2Ca_2Fe_5O_{13}$. The crystal structures are similar to the ones of the Gd-oxides (Reference 18).

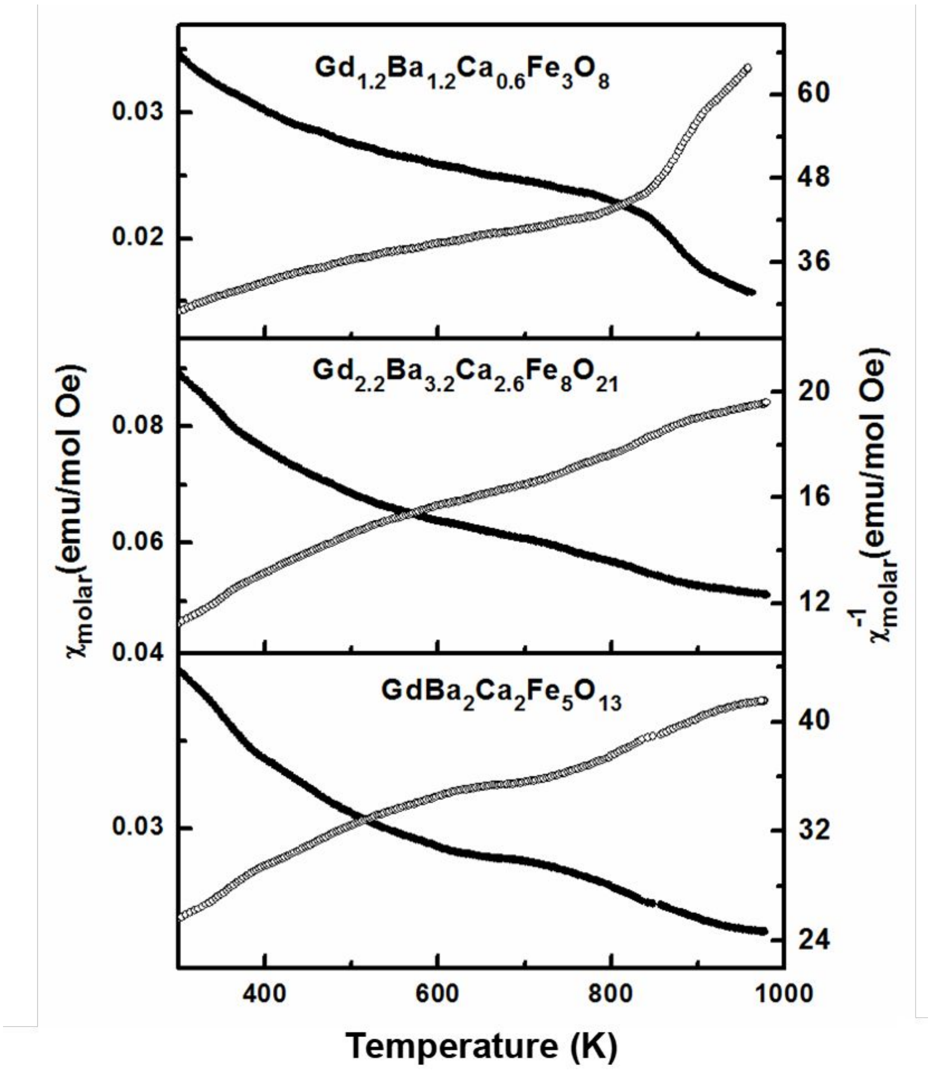

Figure S3. Thermal evolution of the magnetic susceptibility under a magnetic field of 5Oe in the range between 300 and 1000 K of the $Gd_{1.2}Ba_{1.2}Ca_{0.6}Fe_3O_8$, $Gd_{2.2}Ba_{3.2}Ca_{2.6}Fe_8O_{21}$ and $GdBa_2Ca_2Fe_5O_{13}$ oxides.

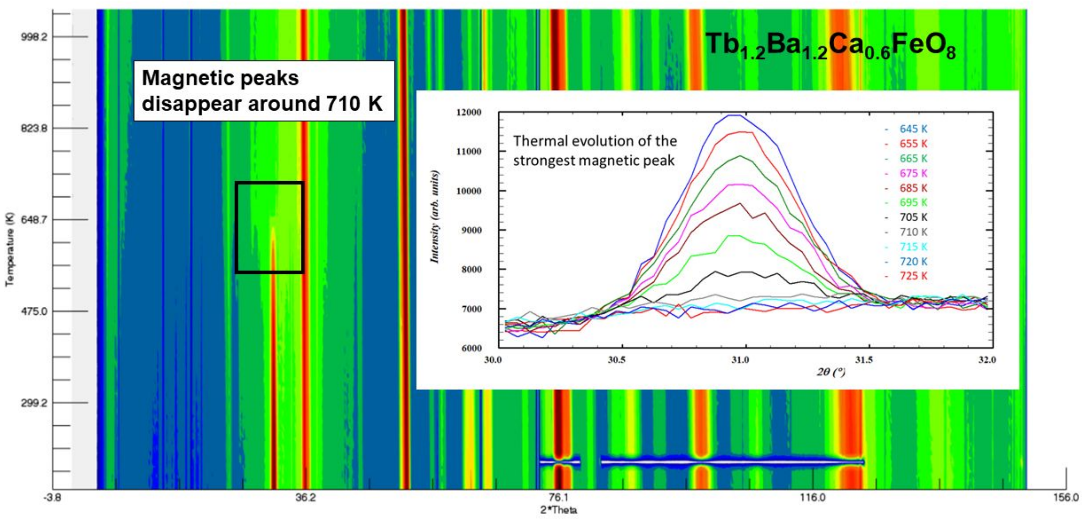

Figure S4. Neutron powder thermo-diffractograms of $Tb_{1.2}Ba_{1.2}Ca_{0.6}Fe_2O_8$ showing the vanishing of magnetic reflections due to the loss of the 3D magnetic order around 710K. The color-code indicates the intensity diffracted, increasing in the sequence: blue<green<yellow<red. The peak highlighted around 31° (2θ) corresponds to the strongest magnetic one $\{110\}_m$ with spacing of 4.52 Å (it appears around 20.3° (2θ) in Figure 3). The inset shows the thermal evolution of this peaks from 650 K to 710 K, temperature at which it vanishes.

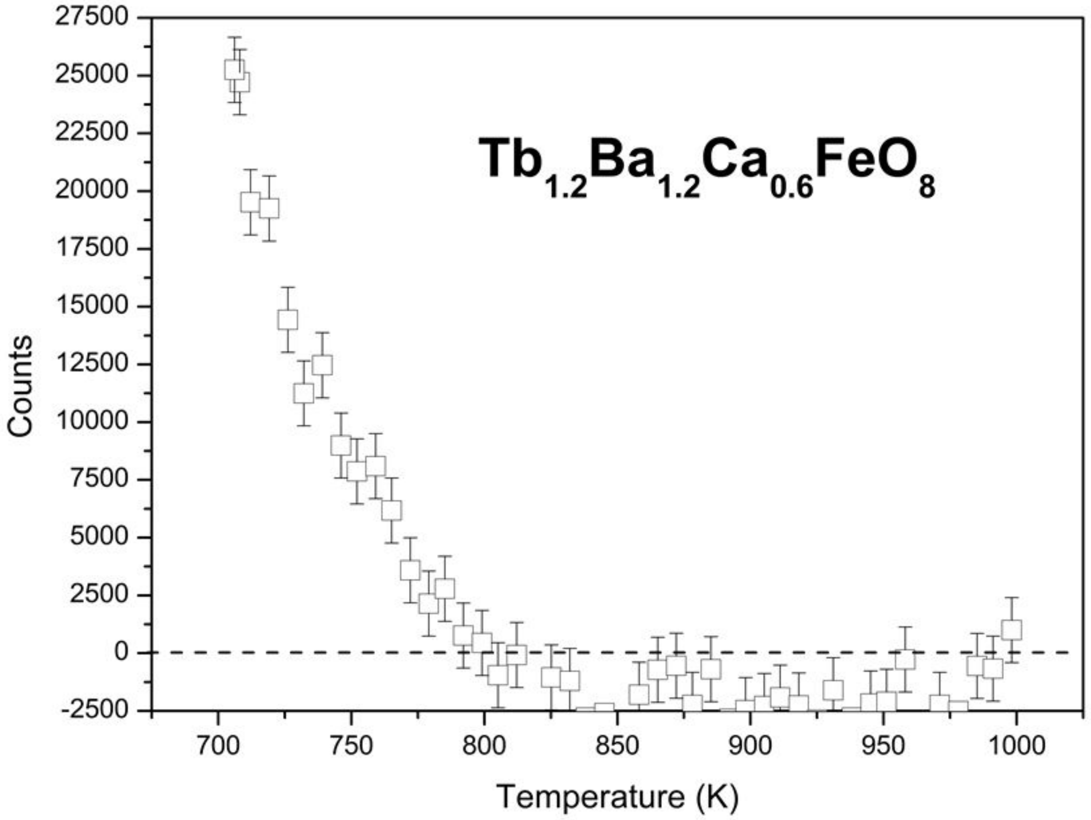

Figure S5. Thermal evolution of the magnetic scattering around the most intense magnetic reflection of the NPD patterns (2θ range between 27.6 and 32.8) of $Tb_{1.2}Ba_{1.2}Ca_{0.6}Fe_2O_8$ between T_{3D} = ~710 K and T_{2D} = ~800 K.

Table S1. Refined crystal structure parameters of $TbBa_2Ca_2Fe_5O_{13}$ at 300 K from the NPD data.

Atom	Wyckoff	X	y	z	B_{iso}	Occupancy
Structural data						
Tb/Ca	4a	0	0	0	0.4(2)	0.55(8)/0.45(8)
Ba	8h	-0.003(2)	0	0.6007(2)	0.6(1)	1
Ca/Tb	8h	-0.016(2)	0	0.6932(2)	0.8(2)	0.77(4)/0.23(4)
Fe(SP)	8h	0.501(1)	0	0.04875(8)	0.83(3)	1
Fe(O2)	8h	0.503(1)	0	0.1523(1)	0.83(3)	1
Fe(T)	4e	0.552(1)	0	0.25	0.83(3)	1
O1	8g	0.25	0.25	0.0393(2)	0.75(3)	1
O2	8g	0.25	0.25	0.9623(2)	0.75(3)	1
O3	8g	0.25	0.25	0.1515(2)	0.75(3)	1
O4	8g	0.25	0.25	0.6582(2)	0.75(3)	1
O5	16j	0.628(2)	0.401(2)	0.25	0.75(3)	0.5
O6	8h	0.508(2)	0	0.0991(2)	0.75(3)	1
O7	8h	0.436(1)	0	0.2073(1)	0.75(3)	1
O8	8h	0.5	0	0	0.75(3)	0.01(2)
Magnetic data						
k = (0,0,0)	Fe(SP)	*3.3(3) μB*	Fe(O)	*4.0(2) μB*	Fe(T)	*4.2(3) μB*
Space Group: Ibmm (#74); a = 5.54769(6) Å b = 5.50949(6) Å c = 38.1917(4) Å						
Rwp = 3.44 %, Rp = 4.42 %, Rexp = 2.17 %, R_B = 5.06 %, χ^2 = 4.14						

Table S2. Refined crystal structure parameters of $TbBa_2Ca_2Fe_5O_{13}$ at 1000 K from the NPD data.

Atom	Wyckoff	x	y	z	B_{iso}	Occupancy
Tb/Ca	4a	0	0	0	2.3(3)	0.55(8)/0.45(8)
Ba	8h	-0.003(3)	0	0.6003(4)	2.4(2)	1
Ca/Tb	8h	-0.012(3)	0	0.6935(2)	1.5(2)	0.77(4)/0.23(4)
Fe(SP)	8h	0.505(2)	0	0.0493(1)	1.88(4)	1
Fe(O2)	8h	0.503(1)	0	0.1528(2)	1.88(4)	1
Fe(T)	4e	0.557(2)	0	0.25	1.88(4)	1
O1	8g	0.25	0.25	0.0395(3)	2.51(4)	1
O2	8g	0.25	0.25	0.9631(3)	2.51(4)	1
O3	8g	0.25	0.25	0.1504(4)	2.51(4)	1
O4	8g	0.25	0.25	0.6578(3)	2.51(4)	1
O5	16j	0.614(4)	0.397(4)	0.25	2.51(4)	0.5
O6	8h	0.511(3)	0	0.0992(3)	2.51(4)	1
O7	8h	0.440(2)	0	0.2077(2)	2.51(4)	1
O8	8h	0.5	0	0.5	2.51(4)	0.02(1)

Space Group: Ibmm (#74); a = 5.6119(1) Å b = 5.5706(1) Å c = 38.7627(7) Å
Rwp = 6.72 %, Rp = 7.98 %, Rexp = 4.76 %, R_B = 6.02 %, χ^2 = 2.81

Table S3. Refined crystal structure parameters of $Tb_{2.2}Ba_{3.2}Ca_{2.6}Fe_8O_{21}$ at 300 K from the NPD data.

Atom	Wyckoff	x	y	z	B_{iso}	Occupancy
Structural data						
Tb/Ca	4a	0	0	0	0.75(3)	0.58(8)/0.42(8)
Ba1	8h	0.003(4)	0	0.064(2)	0.75(3)	1
Ba2	4e	-0.007(3)	0	0.25	0.75(3)	1
Ca/Tb1	8h	0.009(3)	0	0.1218(1)	0.75(3)	0.52(2)/0.47(2)
Ca/Tb2	8h	0.009(3)	0	0.1917(2)	0.75(3)	0.52(2)/0.47(2)
Fe(SP)	8h	0.501(2)	0	0.0290(1)	0.70(2)	1
Fe(O2)	8h	0.501(2)	0	0.0945(1)	0.70(2)	1
Fe(T)	8h	0.546(1)	0	0.1546(1)	0.70(2)	1
Fe(O1)	8h	0.506(1)	0	0.2184(1)	0.70(2)	1
O1	8g	0.25	0.25	0.0244(2)	0.84(3)	1
O2	8g	0.25	0.25	0.9778(2)	0.84(3)	1
O3	8g	0.25	0.25	0.0943(2)	0.84(3)	1
O4	8g	0.25	0.25	0.9044(2)	0.84(3)	1
O5	16j	0.633(2)	0.377(2)	0.1539(2)	0.84(3)	0.5
O6	8g	0.25	0.25	0.2159(2)	0.84(3)	1
O7	8g	0.25	0.25	0.7880(2)	0.84(3)	1
O8	8h	0.523(2)	0	0.0622(2)	0.84(3)	1
O9	8h	0.504(2)	0	0.1290(1)	0.84(3)	1
O10	8h	0.420(2)	0	0.1819(2)	0.84(3)	1
O11	4e	0.524(4)	0	0.25	0.84(3)	1
Magnetic data						
k = (0,1,0)	Fe(SP)	*3.0(3) μB*	Fe(O)	*3.8(2) μB*	Fe(T)	*3.9(2) μB*

Space Group: Ibmm (#74); a = 5.56907(8) Å b = 5.52999(7) Å c = 61.6701(9) Å
Rwp = 4.08 %, Rp = 5.15 %, Rexp = 2.02 %, R_B = 5.48 %, χ^2 = 6.51

Table S4. Refined crystal structure parameters of $Tb_{2.2}Ba_{3.2}Ca_{2.6}Fe_8O_{21}$ at 1000 K from the NPD data.

Atom	Wyckoff	x	y	Z	B_{iso}	Occupancy
Tb/Ca	4a	0	0	0	1.86(8)	0.58(8)/0.42(8)
Ba1	8h	-0.007(7)	0	0.065(3)	1.86(8)	1
Ba2	4e	-0.00(1)	0	0.25	1.86(8)	1
Ca/Tb1	8h	-0.001(7)	0	0.1223(3)	1.86(8)	0.52(2)/0.47(2)
Ca/Tb2	8h	0.010(5)	0	0.1918(2)	1.86(8)	0.52(2)/0.47(2)
Fe(SP)	8h	0.505(4)	0	0.0291(2)	2.12(6)	1
Fe(O2)	8h	0.500(4)	0	0.0930(3)	2.12(6)	1
Fe(T)	8h	0.542(2)	0	0.1567(3)	2.12(6)	1
Fe(O1)	8h	0.499(4)	0	0.2182(2)	2.12(6)	1
O1	8g	0.25	0.25	0.0247(4)	2.42(5)	1
O2	8g	0.25	0.25	0.9680(4)	2.42(5)	1
O3	8g	0.25	0.25	0.0943(2)	2.42(5)	1
O4	8g	0.25	0.25	0.9043(4)	2.42(5)	1
O5	16j	0.624(4)	0.379(4)	0.1531(4)	2.42(5)	0.5
O6	8g	0.25	0.25	0.2168(4)	2.42(5)	1
O7	8g	0.25	0.25	0.7867(4)	2.42(5)	1
O8	8h	0.496(6)	0	0.0615(3)	2.42(5)	1
O9	8h	0.429(4)	0	0.1302(3)	2.42(5)	1
O10	8h	0. 460(4)	0	0.1837(3)	2.42(5)	1
O11	4e	0.51(4)	0	0.25	2.42(5)	1

Space Group: Ibmm (#74); a = 5.6150(1) Å b = 5.57808(8) Å c = 62.382(1) Å
Rwp = 8.32 %, Rp = 6.85 %, Rexp = 4.34 %, R_B = 5.48 %, χ^2 = 3.68

Table S5. Selected interatomic distances in $TbBa_2Ca_2Fe_5O_{13}$ and $Tb_{2.2}Ba_{3.2}Ca_{2.6}Fe_8O_{21}$ at 300 K and 1000 K.

Fe-O Distances at 300 K

	**$Tb_{2.2}Ba_{3.2}Ca_{2.6}Fe_8O_{21}$**		**$Tb_2Ba_2CaFe_5O_{13}$**	
	**Equatorial**	**Apical**	**Equatorial**	**Apical**
**Fe (T)**	1.91(1)<br>2.14(2)	1.82(1)<br>1.60(1)	1.86(1)<br>2.25(1)	2 x 1.754(6)
**Fe (O1)**	2 x 1.990(7)<br>2 x 1.981(8)	1.950(7)<br>2.30(1)		
**Fe (O2)**	2 x 1.966(8)<br>2 x 1.959(8)	1.99(1)<br>2.13(1)	2 x 1.964(5)<br>2 x 1.959(4)	2.03(1)<br>2.132(8)
**Fe (SP)**	2 x 1.986(8)<br>2 x 2.002(8)	1.956(2)	2 x 1.992(5)<br>2 x 1.995(5)	1.923(9)

Fe-O Distances at 1000 K

	**$Tb_{2.2}Ba_{3.2}Ca_{2.6}Fe_8O_{21}$**		**$Tb_2Ba_2CaFe_5O_{13}$**	
	**Equatorial**	**Apical**	**Equatorial**	**Apical**
**Fe (T)**	2.01(1)<br>2.17(3)	1.77(3)<br>1.75(3)	1.93(3)<br>2.24(3)	2 x 1.766(9)
**Fe (O1)**	2 x 2.01(2)<br>2 x 1.97(2)	1.98(1)<br>2.16(3)		
**Fe (O2)**	2 x 1.99(2)<br>2 x 1.98(2)	1.97(3)<br>2.35(5)	2 x 1.989(7)<br>2 x 1.977(7)	2.07(1)<br>2.17(1)
**Fe (SP)**	2 x 2.01(2)<br>2 x 2.01(2)	2.02(3)	2 x 2.03(5)<br>2 x 2.03(8)	1.94(1)
